# Lack of Association Between *Helicobacter pylori* Infection and the Risk of Thyroid Nodule Types: A Multicenter Case-Control Studyin China

**DOI:** 10.3389/fcimb.2021.766427

**Published:** 2021-12-14

**Authors:** Xiao-Song Wang, Xi-Hai Xu, Gang Jiang, Yu-Huan Ling, Tian-Tian Ye, Yun-Wu Zhao, Kun Li, Yu-Ting Lei, Hua-Qing Hu, Ming-Wei Chen, Heng Wang

**Affiliations:** ^1^ Department of Epidemiology and Biostatistics, School of Public Health, Anhui Medical University, Hefei, China; ^2^ The First Affiliated Hospital of Anhui Medical University, Hefei, China; ^3^ Health Management Center, The First Affiliated Hospital of Anhui Medical University, Hefei, China; ^4^ Department of Social Medicine and Health Management, School of Health Management, Anhui Medical University, Hefei, China

**Keywords:** *Helicobacter pylori*, pathogenesis, population, risk, thyroid nodule

## Abstract

The prevalence of *Helicobacter pylori* infection is high worldwide, while numerous research has focused on unraveling the relationship between *H. pylori* infection and extragastric diseases. Although *H. pylori* infection has been associated with thyroid diseases, including thyroid nodule (TN), the relationship has mainly focused on potential physiological mechanisms and has not been validated by large population epidemiological investigations. Therefore, we thus designed a case-control study comprising participants who received regular health examination between 2017 and 2019. The cases and controls were diagnosed *via* ultrasound, while TN types were classified according to the guidelines of the American College of Radiology Thyroid Imaging Reporting and Data System (ACR TI-RADS). Moreover, *H. pylori* infection was determined by C14 urea breath test, while its relationship with TN type risk and severity was analyzed using binary and ordinal logistic regression analyses. A total of 43,411 participants, including 13,036 TN patients and 30,375 controls, were finally recruited in the study. The crude odds ratio (OR) was 1.07 in Model 1 (95% CI = 1.03–1.14) without adjustment compared to the *H. pylori* non-infection group. However, it was negative in Model 2 (OR = 1.02, 95% CI = 0.97–1.06) after being adjusted for gender, age, body mass index (BMI), and blood pressure and in Model 3 (OR = 1.01, 95% CI = 0.97–1.06) after being adjusted for total cholesterol, triglyceride, low-density lipoprotein, and high-density lipoprotein on the basis of Model 2. Control variables, including gender, age, BMI, and diastolic pressure, were significantly correlated with the risk of TN types. Additionally, ordinal logistic regression results revealed that *H. pylori* infection was positively correlated with malignant differentiation of TN (Model 1: OR = 1.06, 95% CI = 1.02–1.11), while Model 2 and Model 3 showed negative results (Model 2: OR = 1.01, 95% CI = 0.96–1.06; Model 3: OR = 1.01, 95% CI = 0.96–1.05). In conclusion, *H. pylori* infection was not significantly associated with both TN type risk and severity of its malignant differentiation. These findings provide relevant insights for correcting possible misconceptions regarding TN type pathogenesis and will help guide optimization of therapeutic strategies for thyroid diseases.

## Introduction

*Helicobacter pylori*, a Gram-negative bacterium that infects the human stomach and accounts for about half of infections worldwide, is difficult to eliminate by the human immune system. Previous studies have shown that *H. pylori* infection rates are on a decline but still high in developing countries (Maluf et al., 2020). *H. pylori* infection has been linked to the pathogenesis of numerous diseases of the upper digestive tract, including gastric cancer, gastric ulcer, and gastritis. Moreover, this infection has also been associated with many extragastric diseases, including hematological, allergic, neurological, ophthalmic, metabolic, and dermatologic diseases (Pero et al., 2019; Gravina et al., 2020). Based on these pieces of evidence, it is necessary to explore the relationship between *H. pylori* infection with other extragastric diseases. Thyroid nodule (TN), which has a prevalence rate of 20%–70% especially in women and the elderly, is a common clinical disorder (Schiaffino et al., 2020). Notably, recent advancements in ultrasonic resolution have also improved the detection rate of TN types (Yu et al., 2020). Previous studies have shown that although TN types are mostly benign, approximately 5%–15% of them may develop into malignancies (Chambara and Ying, 2019). Despite the reliability of the current diagnostics and treatment therapies for TN types, the pathogenesis remains unclear, and several risk factors, including diet, environment, genetics, and abnormal inflammation, have been documented.

Previous studies on the association between *H. pylori* infection and thyroid diseases have mostly focused on thyroid autoimmunity, and the correlation between *H. pylori* infection and thyroid autoimmune diseases, such as Graves disease and Hashimoto thyroiditis, has been reported (Benvenga and Guarneri, 2016; Kohling et al., 2017; Figura et al., 2019). Shen et al. (2013) found that *H. pylori* infection was positively associated with the risk of TN types. Currently, the biological mechanisms that may explain the association mainly include molecular mimicry and dysbiosis (Zhang et al., 2018; Cuan-Baltazar and Soto-Vega, 2020; Docimo et al., 2020). Molecular mimicry theory suggests that there are at least 14 proteins of *H. pylori* antigen epitope similar to the local amino acid sequence of thyroid endogenous proteins (Benvenga and Guarneri, 2016). This structural similarity can trigger an immune cross-reaction and chronic thyroid inflammation, which may explain the reactive hyperplasia induced by *H. pylori* infection (Shi et al., 2013). T helper (Th) cells also play an indirect role in this mechanism, which may co-induce the formation of TN types (Shi et al., 2013; Cuan-Baltazar and Soto-Vega, 2020). In addition, intestinal flora affects endocrine signals through the brain–gut axis, and the intestinal microbial diversity of TN patients is significantly higher than that of healthy population (Zhang et al., 2019). It has been found that *Lactobacillus*, as an important intestinal flora, participates in protecting the thyroid gland from oxidative damage, while *H. pylori* infection has an inhibitory effect on the survival of this bacterium, which may be related to the pathogenesis of TN types (Iino et al., 2018; Zhang et al., 2019). Although these findings have indicated relationships and potential physiological mechanisms, there is still need for validation using large population epidemiological investigations.

Here, we designed a case-control study to verify the relationship between *H. pylori* infection and the risk of TN types. Our results provide valuable insights into the etiology of TN types and are expected to guide future designing of management strategies for this common disorder.

## Methods

### Study Population

This observational study recruited community residents and workers from different organizations and companies who underwent a health examination at the Health Management Center of the First Affiliated Hospital of Anhui Medical University, Hefei, the capital city of Anhui Province, China, between January 2017 and March 2019. The hospital has six centers, located across different regions of the province. The study was approved by the Ethics Committee of the First Affiliated Hospital of Anhui Medical University (number: Quick-PJ 2021-10-28). Classification of TN types and controls was performed *via* ultrasound diagnosis.

The inclusion criteria in this research were mainly based on the type of physical examination items selected by participants, and it is defined as individuals who volunteer for routine physical examinations, including carbon-14 (C14) urea breath test (*H. pylori* infection diagnosis), TN type screening (ultrasonographic diagnosis), height, weight, blood pressure (BP), and blood lipids. We also excluded individuals who have previously undergone thyroidectomy or those who have taken drugs that affect thyroid function, including antithyroid drugs, lithium salts, amiodarone. In addition, individuals who have undergone therapy for *H. pylori* eradication *via* antibiotics, proton pump inhibitor, or bismuth or those who have taken antibiotics within 14 days were also excluded. Finally, a total of 44,682 participants were recruited, and 1,271 individuals were excluded based on the exclusion criteria.

### Data Collection

All participant information was obtained from the physical examination database of the Health Management Center, while *H. pylori* infection was diagnosed using the C14 urea breath test. Participants were first subjected to fasting, for more than 6 h, prior to the C14 breath test. In the C14 breath test, a positive result is defined by a *H. pylori* standard of more than 100 (dpm/mmol), while a range of 0–100 (dpm/mmol) implies a negative result. TN type screening was evaluated using ultrasonographic diagnostic techniques by ultrasonologists with at least 5 years of independent work experience. The TN types were then divided into six grades and classified by American College of Radiology Thyroid Imaging Reporting and Data System (ACR TI-RADS) score. These ACR TI-RADS classification and diagnosis results were used to stratify the patients into the case group, comprising TI-RADS1/2, TI-RADS3, and TI-RADS4/5/6, as the number of patients with TI-RADS1, TI-RADS5, and TI-RADS6 was negligible. The TI-RADS4 group also included TI-RADS4a, TI-RADS4b, and TI-RADS4c, which were not subdivided into groups of different TN types.

Considering the influence of potential confounding factors, we also collected other related information, including gender, age, body mass index (BMI), BP, total cholesterol (TCH), triglyceride (TG), low-density lipoprotein (LDL), and high-density lipoprotein (HDL). These were selected according to previous research findings and expert opinions (Shen et al., 2013; Li et al., 2019; Li et al., 2020). In addition, trained professional nurses systematically collected information on participants’ height, weight, and BP through operation of unified weight and height detector and intelligent BP meter. BMI was calculated as weight divided by height square (kg/m^2^). All other data were obtained from laboratory tests on blood samples, and these were after fasting overnight or over 8 h.

### Statistical Analysis

Statistical analyses were performed using SPSS software version 22.0, and all quantitative variables were presented as means ± standard deviations, while qualitative variables were expressed as quantity and percentages. Rates among different groups were compared using the chi-square test, while differences between quantitative variables were determined using a Student’s *T*-test or Mann–Whitney U test. The relationship between *H. pylori* infection and the risk of TN types was determined using a binary logistic regression. We also calculated tolerance and variance inflation factor for model diagnosis in order to exclude multicollinearity among different independent variables. Multicollinearity was considered to be positive if the tolerance was less than 0.1 or the variance inflation factor was more than 10. In addition, we performed ordinal logistic regression to identify the potential association between *H. pylori* infection and the severity of TN types. Data followed by *P* < 0.05 were considered statistically significant.

## Results

### Participant Characteristics

A total of 43,411 participants, of whom 13,036 and 30,375 were TN patients and controls, respectively, were included in this study (**Table 1**). Among them, 17,697 subjects (40.8%) were positive for *H. pylori* infection, including 5,461 TN cases and 12,236 controls. Notably, *H. pylori* positivity rate was significantly higher in the TN types than that in control groups (*P* = 0.002). Similarly, the average age and the proportion of female subjects were significantly higher in the TN type group relative to those in the controls (each *P* < 0.001). In addition, all other indicators, namely, BMI, systolic pressure, diastolic pressure, TCH, TG, LDL, and HDL, were significantly higher in TN type groups than those in controls (all *P* < 0.001).

**Table 1:** Participant characteristics.

Characteristics	Total	TN types	Controls	*P*
Samples, n	43,411	13,036	30,375	–
Women, n	18,096	6,625	11,471	<0.001
Age, years	45.68 ± 14.08	52.64 ± 14.04	42.69 ± 13.00	<0.001
BMI, kg/m^2^	23.60 ± 3.25	23.85 ± 3.17	23.50 ± 3.27	<0.001
SP, mmHg	126.37 ± 18.23	129.99 ± 19.61	124.81 ± 17.38	<0.001
DP, mmHg	76.82 ± 11.95	77.82 ± 12.00	76.39 ± 11.91	<0.001
TCH, mmol/L	4.65 ± 0.90	4.76 ± 0.95	4.60 ± 0.88	<0.001
TG, mmol/L	1.61 ± 1.41	1.64 ± 1.48	1.59 ± 1.38	<0.001
LDL-C, mmol/L	2.68 ± 0.79	2.76 ± 0.82	2.65 ± 0.77	<0.001
HDL-C, mmol/L	1.38 ± 0.38	1.40 ± 0.39	1.37 ± 0.38	<0.001
14 C-urea breath test, n (+)	17,697	5,461	12,236	0.002

TN, thyroid nodule; BMI, body mass index; SP, systolic pressure; DP, diastolic pressure; TCH, total cholesterol; TG, triglyceride; LDL, low-density lipoprotein; HDL, high-density lipoprotein.

### Prevalence of Thyroid Nodule Types Between *H. pylori-*Infected and Non-Infected Groups

**Figure 1:**
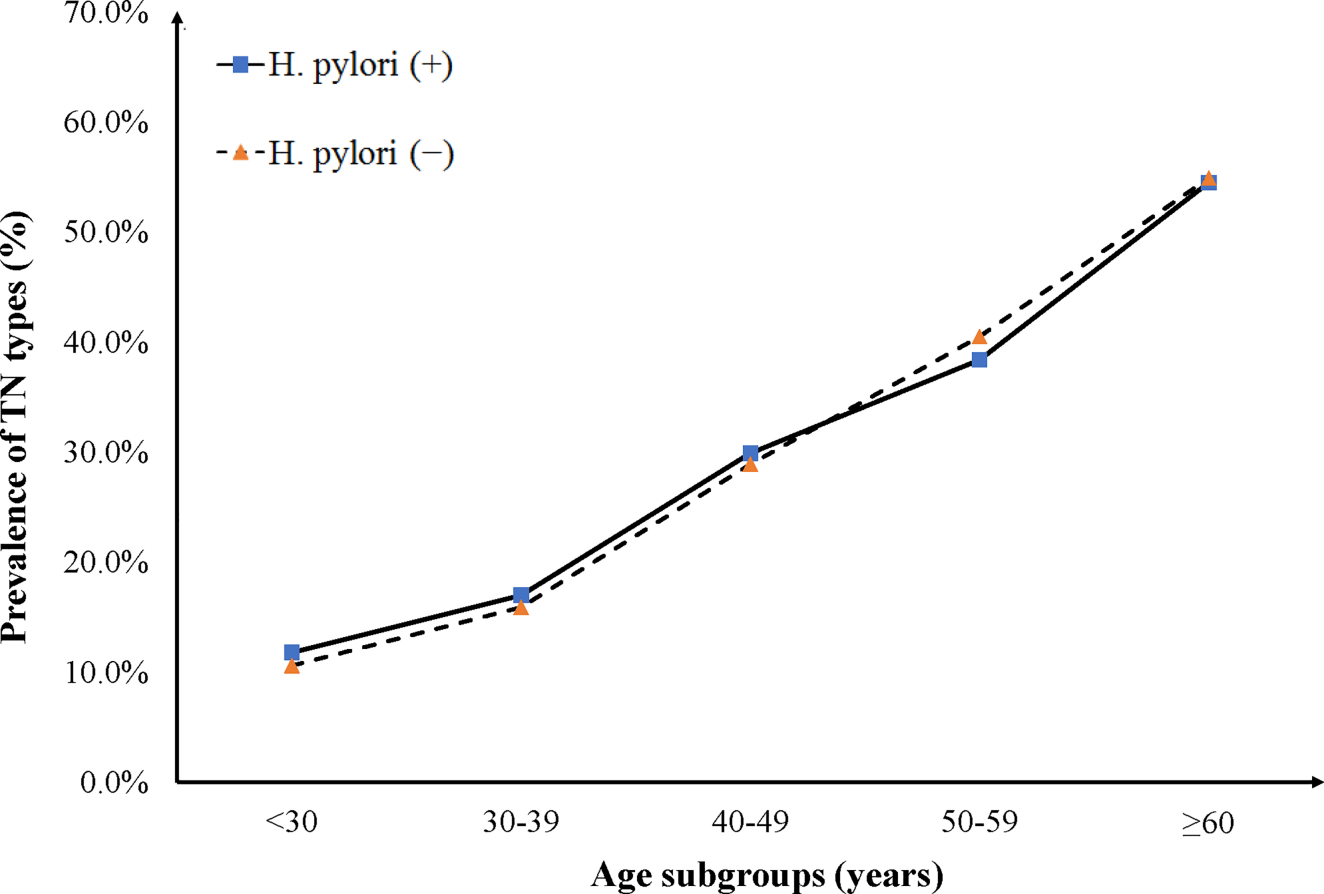
The prevalence of TN types increased with age in both *H. pylori* (+) and *H. pylori* (-) groups. Among the five age ranges, only in the 50-59 years subgroup, the prevalence of TN types in the *H. pylori* (+) group was statistically lower than that in the *H. pylori* (-) group. However, in other age subgroups, no significant difference was found in the prevalence of TN types between *H. pylori* (+) and *H. pylori* (-) groups.

**Figure 2:**
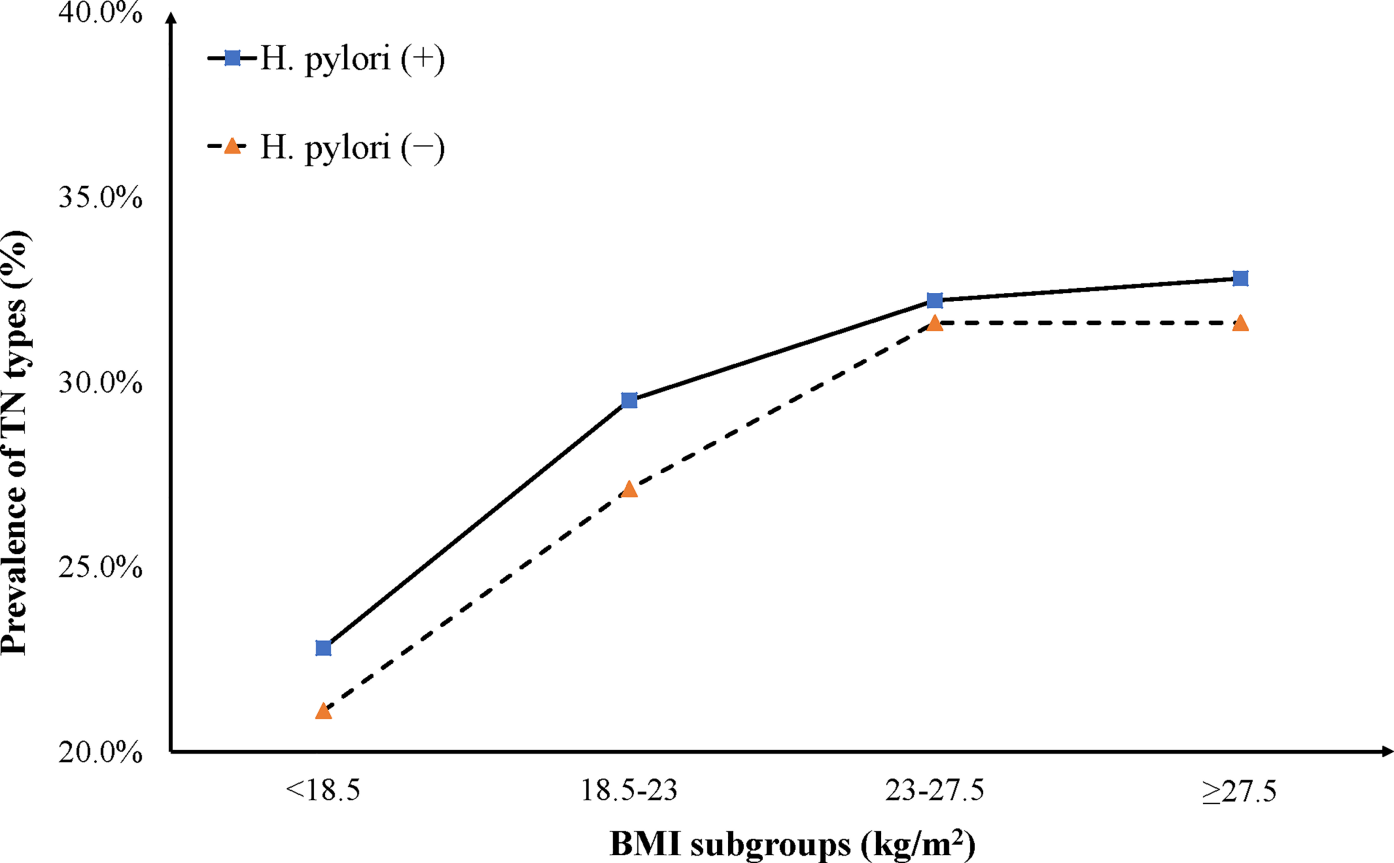
The prevalence of TN types increased slowly with the increase of BMI. Among the four BMI ranges, only in the 18.5-23 kg/m^2^ subgroup, the prevalence of TN types in the *H. pylori* (+) group was statistically higher than that in the *H. pylori* (-) group, and no significant difference was found in the prevalence of TN types between *H. pylori* (+) and *H. pylori* (-) groups in other BMI subgroups

We compared the prevalence of TN types between subjects infected with *H. pylori* and non-infected counterparts across different subgroups, including gender, age, and BMI. Results from the female subgroup indicated a significantly higher prevalence in the infected group than that in the non-infected group (*P* < 0.001), and this was slightly different from the male subgroup (*P* = 0.159). On the other hand, the prevalence of TN types increased in both age and BMI subgroups, although no significant differences were observed between *H. pylori*-infected and non-infected groups. Notably, significant differences in the prevalence of TN types were only found in age range (50–59 years, *P* = 0.030) (**Figure 1**) and BMI (18.5–23 kg/m^2^, *P* = 0.001) (**Figure 2**).

### Correlation Between *H. pylori* Infection and the Risk of Thyroid Nodule Types

We adopted three binary logistic regression models to calculate odds ratios (ORs) and depict the correlation between *H. pylori* infection and the risk of TN types (**Table 2**). Results from Model 1, which employed the univariate logistic regression algorithm, revealed that the crude odds for TN types was 7% (OR = 1.07, 95% CI = 1.03–1.14), and this was positively correlated with *H. pylori* non-infection group. However, results from Model 2 revealed a negative correlation adjusted for sex, age, BMI, systolic pressure, and diastolic pressure (OR = 1.02, 95% CI = 0.97–1.06). On the other hand, Model 3 revealed stable and negative results (OR = 1.01, 95% CI = 0.97–1.06), adjusted for additional control variables, including TCH, TG, LDL-C, and HDL-C, on the basis of Model 2. In Model 2, control variables, such as gender, age, BMI, and diastolic pressure, were significantly associated with the risk of TN types (gender: OR = 2.06, 95% CI = 1.96–2.16; age: OR = 1.05, 95% CI = 1.05–1.06; BMI: OR = 1.04, 95% CI = 1.03–1.05; diastolic pressure: OR > 1.00, 95% CI = 1.00–1.01). Similarly, in Model 3, gender, age, BMI, and diastolic pressure were also significantly associated with the risk of TN types (gender: OR = 2.12, 95% CI = 2.01–2.23; age: OR = 1.05, 95% CI = 1.05–1.06; BMI: OR = 1.03, 95% CI = 1.02–1.04; diastolic pressure: OR > 1.00, 95% CI = 1.00–1.01). However, we found no statistically significant correlation between other control variables and the risk of TN types.

**Table 2:** OR for the association between *H. pylori* infection and the risk of TN types.

C14 test	TN types	Controls	Model 1	Model 2	Model 3
	n (%)	n (%)	OR (95% CI)	OR (95% CI)	OR (95% CI)
*H. pylori* (−)	7,575 (58.1%)	18,139 (59.7%)	1.00 (reference)	1.00 (reference)	1.00 (reference)
*H. pylori* (+)	5,461 (41.9%)	12,236 (40.3%)	1.07 (1.03–1.14)	1.02 (0.97–1.06)	1.01 (0.97–1.06)
*P* value	–	–	0.002^*^	0.505	0.597

Model 1: unadjusted; Model 2: adjusted for gender, age, BMI, systolic, diastolic; Model 3: Model 2 + adjusted for TCH, TG, LDL, HDL.

TN, thyroid nodule; C14 test, carbon-14 urea breath test; CI, confidence interval; BMI, body mass index; SP, systolic pressure; DP, diastolic pressure; TCH, total cholesterol; TG, triglyceride; LDL, low-density lipoprotein; HDL, high-density lipoprotein.

*P value is considered statistically significant.

In the binary logistic analysis results, control variables with *P* value <0.001, including gender, age, and BMI, were analyzed by stratification in order to control for potential confounding bias (**Table 3**). The three models revealed no statistically significant correlation between *H. pylori* infection and the risk of TN types in the male subgroup (Model 1: OR = 1.04, 95% CI = 0.98–1.08; Model 2: OR = 1.01, 95% CI = 0.95–1.08; Model 3: OR = 1.01, 95% CI = 0.95–1.07). However, in the female subgroup, Model 1 revealed a positive correlation between *H. pylori* infection and the risk of TN types (Model 1: OR = 1.12, 95% CI = 1.05–1.20), whereas both Model 2 and Model 3 revealed a negative correlation (Model 2: OR = 1.02, 95% CI = 0.96–1.10; Model 3: OR = 1.02, 95% CI = 0.95–1.09). In age subgroups, with the increase of age, ORs of all three models showed a downward trend, but the results still showed that *H. pylori* infection was not a risk factor for TN types. With regard to BMI, changes in ORs between different layers were stable, although subgroup 18.5–23 kg/m^2^ showed a relatively high OR, which was accompanied by a negative overall correlation.

**Table 3:** Subgroup analysis of *H. pylori* infection and the risk of TN types.

Subgroups	Samples	*H. pylori*		Model 1	Model 2	Model 3
	n (%)	TN types	Controls	OR (95% CI)	OR (95% CI)	OR (95% CI)
Gender						
Male	25,307 (58.3%)	6,409 (49.2%)	18,898 (62.2%)	1.04 (0.98–1.10)	1.01 (0.95–1.08)	1.01 (0.95–1.07)
Female	18,096 (41.7%)	6,625 (50.8%)	11,471 (37.8%)	1.12 (1.05–1.20)^*^	1.02 (0.96–1.10)	1.02 (0.95–1.09)
Age						
<30	5,753 (13.3%)	635 (4.9%)	5,118 (16.8%)	1.13 (0.95–1.34)	1.16 (0.97–1.39)	1.19 (0.99–1.43)
30–39	10,708 (24.7%)	1,746 (13.4%)	8,962 (29.5%)	1.09 (0.98–1.21)	1.10 (0.99–1.23)	1.11 (0.99–1.24)
40–49	10,475 (24.1%)	3,070 (23.6%)	7,405 (24.4%)	1.05 (0.96–1.14)	1.06 (0.97–1.16)	1.04 (0.95–1.15)
50–59	9,464 (21.8%)	3,746 (28.7%)	5,718 (18.8%)	0.91 (0.94–0.99)^*^	0.92 (0.85–1.01)	0.92 (0.84–1.00)
≥60	7,010 (16.1%)	3,839 (29.4%)	3,171 (10.4%)	0.98 (0.89–1.08)	0.96 (0.87–1.06)	0.96 (0.87–1.06)
BMI						
<18.5	1,710 (4.2%)	372 (3.1%)	1,338 (4.7%)	1.10 (0.87–1.39)	1.03 (0.80–1.33)	0.99 (0.76–1.29)
18.5–23	16,370 (40.2%)	4,593 (37.7%)	11,777 (41.3%)	1.13 (1.05–1.21)^*^	1.06 (0.98–1.14)	1.06 (0.98–1.14)
23–27.5	18,059 (44.4%)	5,753 (47.2%)	12,306 (43.2%)	1.02 (0.96–1.09)	0.98 (0.92–1.05)	0.97 (0.90–1.04)
≥27.5	4,557 (11.2%)	1,464 (12.0%)	3,093 (10.8%)	1.06 (0.94–1.20)	1.03 (0.90–1.18)	1.05 (0.92–1.21)

Model 1: unadjusted; Model 2: adjusted for gender, age, BMI, SP, and DP; Model 3: Model 2 + adjusted for TCH, TG, LDL, and HDL.

TN, thyroid nodule; CI, confidence interval; BMI, body mass index; SP, systolic pressure; DP, diastolic pressure; TCH, total cholesterol; TG, triglyceride; LDL, low-density lipoprotein; HDL, high-density lipoprotein.

*P value is considered statistically significant.

### Correlation Between *H. pylori* Infection and the Risk of Thyroid Nodule Type Severity

We performed ordinal logistic regression in order to analyze a potential relationship between *H. pylori* infection and malignant tendency of TN types (**Table 4**). Summarily, we divided patients with different TN types into three grades, according to the ultrasound diagnosis results, then finally divided them into four grades, including normal controls. Results from Model 1 revealed a positive relationship (OR = 1.06, 95% CI = 1.02–1.11), whereas both Model 2 and Model 3 revealed negative results even after inclusion of different control variables (Model 2: OR = 1.01, 95% CI = 0.96–1.06; Model 3: OR = 1.01, 95% CI = 0.96–1.05). The positive correlation observed in Model 1 might be attributed to the influence of various confounding factors.

**Table 4:** Ordinal logistic regression analysis of the *H. pylori* infection and the severity of TN types.

C14 test	The classification of TN types, n (%)				Model 1	Model 2	Model 3
	Negative	TI-RADS ≤ 2	TI-RADS3	TI-RADS≥4	OR (95% CI)	OR (95% CI)	OR (95% CI)
*H. pylori* (−)	18,139 (59.7%)	1,603 (56.2%)	5,556 (58.8%)	416 (57.1%)	1.00 (reference)	1.00 (reference)	1.00 (reference)
*H. pylori* (+)	12,236 (40.3%)	1,251 (43.8%)	3,897 (41.2%)	313 (42.9%)	1.06 (1.02–1.11)	1.01 (0.96–1.06)	1.01 (0.96–1.05)
*P* value	–	–	–	–	0.005^*^	0.695	0.848

Model 1: unadjusted; Model 2: adjusted for sex, age, BMI, systolic, diastolic; Model 3: Model 2 + adjusted for TCH, TG, LDL, and HDL.

TN, thyroid nodule; C14 test, carbon-14 urea breath test; CI, confidence interval; BMI, body mass index; SP, systolic pressure; DP, diastolic pressure; TCH, total cholesterol; TG, triglyceride; LDL, low-density lipoprotein; HDL, high-density lipoprotein.

*P value is considered statistically significant.

## Discussion

Although previous reports have shown that *H. pylori* infection is involved in various thyroid diseases, very few of these studies analyzed large sample sizes. Results of the present study, comprising a population-based case-control study of community residents and workers from different organizations or companies, indicated that *H. pylori* infection was not significantly associated with the risk of TN types, which were in contrast with previous reports (Shen et al., 2013). We validated this lack of significant association through ordinal logistic regression analyses.

The findings of our study, as well as those from published literature, indicated that the theoretical mechanisms underlying the possible association between *H. pylori* infection and the risk of TN types are not rigorous. Molecular mimicry is considered a possible mechanism underlying the relationship between *H. pylori* infection and pathophysiology mechanisms of thyroid diseases. In fact, previous evidence has suggested that infection-induced chronic inflammation could be a crucial cause of TN types, which may also be related to the structural similarity between *H. pylori* epitope antigen and thyroid autoantigen (Yu et al., 2019; Liu et al., 2020). It has been reported that 14 proteins of *H. pylori* antigen epitope are similar to thyroid endogenous proteins, including the segments of human thyrotropin receptor, thyroid autoantigen, and sodium iodide symporter (Benvenga and Guarneri, 2016). Moreover, previous studies have revealed the structural similarities between *H. pylori* epitope and H-K-ATPase in the thyroid gland as well. Furthermore, immune responses, induced by *H. pylori* infection, have been shown to indirectly cause Th1 activation and apoptosis and promote secretion of pro-inflammatory cytokines, including tumor necrosis factor-alpha (TNF-α) and interferon-gamma (INF-γ), thereby causing thyroid tissue injury and inflammation (Cuan-Baltazar and Soto-Vega, 2020). However, molecular mimicry has only been used to explain the autoimmune thyroid pathophysiology caused by *H. pylori* infection; thus, it may not explain the comprehensive induction of TN type development. Another possible theoretical mechanism is dysbiosis. Although the composition of gut microbiome of TN patients differs from that of healthy controls (Zhang et al., 2019; Docimo et al., 2020), there is no definite evidence to affirm that *H. pylori* infection can directly induce such differences in the gut microbiome and the dysbiosis clearly causes TN types.

Results of the present study further indicated that control variables, including gender, age, and BMI, were significantly correlated with the risk of TN types, which was consistent with findings from previous studies (Kwong et al., 2015; Zheng, 2015; Jasim et al., 2020). Collectively, it may imply that the results of this study are reliable and better reflect the association between *H. pylori* infection and the risk of TN types. Based on our results, we can consider that female, increase in age, and BMI were all risk factors for TN types, while the potential interactions in effects across different confounding factors may also have an impact on the risk of TN types. Therefore, it is possible that the influence of these confounders or selection bias in study population might contribute to false positive results of *H. pylori* infection associated with TN types that has been previously reported. Our results also suggested that higher diastolic pressure might be a risk factor for TN types. This is similar to the findings of Li et al. (2020) who reported that higher systolic pressure was positively correlated with increased risk of TN types in a female cross‐sectional study. The mechanism may be that *H. pylori* infection often induces an increase in serum fibrinogen, which interferes with the release of nitric oxide from vascular endothelium. This mechanism tends to inhibit normal relaxation of blood vessels, while vasoconstriction is the main factor leading to increased diastolic BP (Migneco et al., 2003). However, the potential biological mechanisms underlying the observed association between diastolic pressure and the risk of TN types necessitate further exploration using animal experiments or cohort studies.

This study had some limitations. Firstly, we also compiled family history including past diseases suffered and lifestyle habits of the study population, which may be potential confounding factors. However, the information was incomplete due to low response rate of supplementary questionnaires; thus, it was not finally included in the regression models. In addition, considering the feasibility of the study implementation, there may be other potential confounding factors such as radiation exposure to head and neck that have not been collected as well, and these factors may also have an impact on the results. Secondly, there may still be potential selection bias in study population. Patients with a high risk for malignant TN types, diagnosed with TI-RADS5 or TI-RADS6, are few in this research. This might be attributed to the fact that residents and workers with higher TI-RADS grades may be more inclined to directly choosing hospital treatment, as opposed to receiving physical examination at a health management center, though residents and workers in the surveyed areas had a high prevalence of regular health screenings. Moreover, Anhui is one of the most densely populated provinces in eastern China. Actually, the included participants in this research were mainly from urban areas of Anhui, and the majority was from the capital city, Hefei. These factors may also affect the representation of the study population. Finally, this study employed a case-control survey design; thus, it was difficult to analyze the causality between potential risk factors and TN types. Therefore, further better designed studies are needed to validate these results.

In summary, we found no significant correlation between *H. pylori* infection and neither TN type risk nor the degree of its malignant differentiation, indicating that *H. pylori* infection neither promotes nor induces TN types. Therapies for eliminating *H. pylori* are not recommended for TN patients as an independent measure to reduce the risk of malignant differentiation. Further explorations, using large prospective studies, are needed to fully elucidate the association between *H. pylori* infection and more thyroid disorders.

## Data Availability Statement

The original contributions presented in the study are included in the article/supplementary material. Further inquiries can be directed to the corresponding author.

## Ethics Statement

The studies involving human participants were reviewed and approved by The Ethics Committee of the First Affiliated Hospital of Anhui Medical University. Written informed consent for participation was not required for this study in accordance with the national legislation and the institutional requirements.

## Author Contributions

HW: Research design. X-SW: Article drafting and data analysis. GJ: Data collation and analysis. Y-HL, T-TY, and Y-WZ: Data collation. X-HX, KL, and Y-TL: Data collection. M-WC and H-QH: Revisions of article. All authors contributed to the article and approved the submitted version.

## Funding

This study was supported by the National Key R&D Program of China (2020YFC2006500, 2020YFC2006502).

## Conflict of Interest

The authors declare that the research was conducted in the absence of any commercial or financial relationships that could be construed as a potential conflict of interest.

## Publisher’s Note

All claims expressed in this article are solely those of the authors and do not necessarily represent those of their affiliated organizations, or those of the publisher, the editors and the reviewers. Any product that may be evaluated in this article, or claim that may be made by its manufacturer, is not guaranteed or endorsed by the publisher.

## References

[B1] Benvenga S. Guarneri F. (2016). Molecular Mimicry and Autoimmune Thyroid Disease. Rev. Endocr. Metab. Disord. 17 (4), 485–498. doi: 10.1007/s11154-016-9363-2 27307072

[B2] Chambara N. Ying M. (2019). The Diagnostic Efficiency of Ultrasound Computer-Aided Diagnosis in Differentiating Thyroid Nodules: A Systematic Review and Narrative Synthesis. Cancers (Basel) 11 (11), 1759. doi: 10.3390/cancers11111759 PMC6896127 31717365

[B3] Cuan-Baltazar Y. Soto-Vega E. (2020). Microorganisms Associated to Thyroid Autoimmunity. Autoimmun. Rev. 19 (9):102614. doi: 10.1016/j.autrev.2020.102614 32663624

[B4] Docimo G. Cangiano A. Romano R. M. Pignatelli M. F. Offi C. Paglionico V. A. (2020). The Human Microbiota in Endocrinology: Implications for Pathophysiology, Treatment, and Prognosis in Thyroid Diseases. Front. Endocrinol. (Lausanne) 11:586529. doi: 10.3389/fendo.2020.586529 33343507 PMC7746874

[B5] Figura N. Di Cairano G. Moretti E. Iacoponi F. Santucci A. Bernardini G. (2019). Helicobacter Pylori Infection and Autoimmune Thyroid Diseases: The Role of Virulent Strains. Antibiotics (Basel) 9 (1), 12. doi: 10.3390/antibiotics9010012 PMC7167994 31906000

[B6] Gravina A. G. Priadko K. Ciamarra P. Granata L. Facchiano A. Miranda A. (2020). Extra-Gastric Manifestations of Helicobacter Pylori Infection. J. Clin. Med. 9 (12):3887. doi: 10.3390/jcm9123887 PMC7761397 33265933

[B7] Iino C. Shimoyama T. Chinda D. Arai T. Chiba D. Nakaji S. (2018). Infection of Helicobacter Pylori and Atrophic Gastritis Influence Lactobacillus in Gut Microbiota in a Japanese Population. Front. Immunol. 9:712. doi: 10.3389/fimmu.2018.00712 29681906 PMC5897428

[B8] Jasim S. Baranski T. J. Teefey S. A. Middleton W. D. (2020). Investigating the Effect of Thyroid Nodule Location on the Risk of Thyroid Cancer. Thyroid 30 (3), 401–407. doi: 10.1089/thy.2019.0478 31910102 PMC7074921

[B9] Kohling H. L. Plummer S. F. Marchesi J. R. Davidge K. S. Ludgate M. (2017). The Microbiota and Autoimmunity: Their Role in Thyroid Autoimmune Diseases. Clin. Immunol. 183, 63–74. doi: 10.1016/j.clim.2017.07.001 28689782

[B10] Kwong N. Medici M. Angell T. E. Liu X. Marqusee E. Cibas E. S. (2015). The Influence of Patient Age on Thyroid Nodule Formation, Multinodularity, and Thyroid Cancer Risk. J. Clin. Endocrinol. Metab. 100 (12), 4434–4440. doi: 10.1210/jc.2015-3100 26465395 PMC4667162

[B14] Liu X. Z. Wang J. M. Ji Y. X. Zhao D. B. (2020). Monocyte-To-High-Density Lipoprotein Cholesterol Ratio is Associated With the Presence and Size of Thyroid Nodule Irrespective of the Gender. Lipids Health Dis. 19 (1), 36. doi: 10.1186/s12944-020-1196-z 32164741 PMC7069177

[B12] Li H. Wang Z. Liu J. S. Zou B. S. Chen H. R. Xu Z. (2020). Association Between Breast and Thyroid Lesions: A Cross-Sectional Study Based on Ultrasonography Screening in China. Thyroid 30 (8), 1150–1158. doi: 10.1089/thy.2019.0184 32148169

[B13] Li L. Ying Y. Zhang C. Wang W. Li Y. Feng Y. (2019). Bisphenol A Exposure and Risk of Thyroid Nodules in Chinese Women: A Case-Control Study. Environ. Int. 126, 321–328. doi: 10.1016/j.envint.2019.02.026 30825751

[B15] Maluf S. Salgado J. V. Cysne D. N. Camelo D. M. F. Nascimento J. R. Maluf B. V. T. (2020). Increased Glycated Hemoglobin Levels in Patients With Helicobacter Pylori Infection Are Associated With the Grading of Chronic Gastritis. Front. Immunol. 11:2121. doi: 10.3389/fimmu.2020.02121 33013895 PMC7511518

[B16] Migneco A. Ojetti V. Specchia L. Franceschi F. Candelli M. Mettimano M. (2003). Eradication of Helicobacter Pylori Infection Improves Blood Pressure Values in Patients Affected by Hypertension. Helicobacter 8 (6), 585–589. doi: 10.1111/j.1523-5378.2003.00180.x 14632672

[B17] Pero R. Brancaccio M. Laneri S. Biasi M. G. Lombardo B. Scudiero O. (2019). A Novel View of Human Helicobacter Pylori Infections: Interplay Between Microbiota and Beta-Defensins. Biomolecules 9 (6), 237. doi: 10.3390/biom9060237 PMC6627275 31216758

[B18] Schiaffino S. Serpi F. Rossi D. Ferrara V. Buonomenna C. Ali M. (2020). Reproducibility of Ablated Volume Measurement Is Higher With Contrast-Enhanced Ultrasound Than With B-Mode Ultrasound After Benign Thyroid Nodule Radiofrequency Ablation-A Preliminary Study. J. Clin. Med. 9 (5), 1504. doi: 10.3390/jcm9051504 PMC7291258 32429487

[B19] Shen Z. Qin Y. Liu Y. Lu Y. Munker S. Chen L. (2013). Helicobacter Pylori Infection is Associated With the Presence of Thyroid Nodules in the Euthyroid Population. PloS One 8 (11), e80042. doi: 10.1371/journal.pone.0080042 24244604 PMC3823768

[B20] Shi W. J. Liu W. Zhou X. Y. Ye F. Zhang G. X. (2013). Associations of Helicobacter Pylori Infection and Cytotoxin-Associated Gene A Status With Autoimmune Thyroid Diseases: A Meta-Analysis. Thyroid 23 (10), 1294–1300. doi: 10.1089/thy.2012.0630 23544831

[B21] Yu J. Deng Y. Liu T. Zhou J. Jia X. Xiao T. (2020). Lymph Node Metastasis Prediction of Papillary Thyroid Carcinoma Based on Transfer Learning Radiomics. Nat. Commun. 11 (1), 4807. doi: 10.1038/s41467-020-18497-3 32968067 PMC7511309

[B22] Yu S. T. Ge J. N. Li R. C. Wei Z. G. Sun B. H. Jiang Y. M. (2019). Is Epstein-Barr Virus Infection Associated With Thyroid Tumorigenesis?-A Southern China Cohort Study. Front. Oncol. 9:312. doi: 10.3389/fonc.2019.00312 31134145 PMC6524691

[B23] Zhang J. Zhang F. Zhao C. Xu Q. Liang C. Yang Y. (2018). Dysbiosis of the Gut Microbiome is Associated With Thyroid Cancer and Thyroid Nodules and Correlated With Clinical Index of Thyroid Function. Endocrine 64 (3), 564–574. doi: 10.1007/s12020-018-1831-x 30584647

[B11] Zheng L. Yan W. Kong Y. Liang P. Mu Y. (2015). An Epidemiological Study of Risk Factors of Thyroid Nodule and Goiter in Chinese Women. Int. J. Clin. Exp. Med. 8 (7), 11379–11387. doi: 10.3390/ijerph120911608 26379953 PMC4565336

